# Two-hole assisted phaco-chop technique: a more efficient method for safe nucleofractis vertical chopping

**DOI:** 10.1007/s10792-021-01872-4

**Published:** 2021-05-04

**Authors:** Naomi Miyamoto, Shoko Kiritoshi, Rinko Akamime, Masayuki Akimoto

**Affiliations:** Department of Ophthalmology, Osaka Red Cross Hospital, 5-30 Fudegasakicho, Tennoji-ku, Osaka, 543-8555 Japan

**Keywords:** Phaco-chop, Nucelofractis, Two-hole, Cataracts

## Abstract

**Purpose:**

To evaluate the use of the two-hole technique in augmenting the efficiency of surgeons-in-training when performing the phaco-chop technique. We hypothesized that drilling two holes in opposite angles to each other adjacent to the capsulorhexis would mimic a new lens equator. The phaco-tip and the chopper can be inserted into these holes at appropriate depths and may sandwich and fracture the lens more easily than conventional methods.

**Methods:**

The two-hole technique described above was performed by three first-year surgeons before they performed the standard phaco-chop technique. We collected data of their first 8 cases and analyzed a total of 64 cases: 16, divide-and-conquer; 24, two-hole method; 24, phaco-chop. The main outcome measures included the cumulative dissipated energy (CDE) and case ultrasound time (UST) with different phacoemulsification techniques.

**Results:**

The young practicing surgeons eventually performed the standard phaco-chop more safely after repeated practice using the two-hole method. The drilling of holes enabled deep and effortless impaling of the nucleus. Although there was no significant difference in the CDE among the techniques, there was a significant difference in the UST (*P* < 0.05).

**Conclusion:**

The two-hole method enabled surgeons-in-training to acquire standard phaco-chop skills more efficiently. However, further studies with higher statistical power will be needed to validate these findings. Additionally, a variation of this technique, the four-hole method, is applicable even for experienced surgeons in cases of a hardened nucleus.

**Supplementary Information:**

The online version contains supplementary material available at 10.1007/s10792-021-01872-4.

## Introduction

A successful nuclear fracture is a crucial step in the phacoemulsification technique. Divide-and-conquer, presented by Gimbel in 1991 [1], is a standard and widely accepted technique used by cataract surgeons. Phaco-chop, developed by Nagahara in 1993 [2], is considered one of the most effective methods for nuclear fracturing and requires less time for ultrasound sculpting [3–5]. Some variants, such as the two-dimensional horizontal chop or three-dimensional vertical chop, have been developed and accepted for use. The vertical chop method enables deep nuclear penetration and is particularly effective for the management of a hardened nucleus. Many modified techniques for hard cataracts have been described wherein the central part of the nucleus is drilled (or dug) to facilitate its disassembly [6–8].

Although frequently compared with the divide-and-conquer method, phaco-chop reduces the ultrasound energy required for nucleofractis. However, young surgeons need extensive efforts and training to shift from the divide-and-conquer technique to phaco-chop successfully. Usually, phaco-chop is taught to new surgeons partially, for example, only in the second and third chopping.

The correct placement of the chopper is one of the difficult maneuvers in phaco-chop. Surgeons-in-training tend to lacerate the capsulorhexis when they attempt to divide the nucleus from the periphery. If the pupil size is small, such as in patients with intraoperative floppy iris syndrome (IFIS), the capsulorhexis tends to be inadequate. This increases the risk of lacerating the capsulorhexis or pupillary margin by the chopper.

In cases where there is an excessively soft or a hard nucleus, embedding both a phaco-tip and a chopper to a depth sufficient to sandwich and chop the lens may also be difficult, especially in the first chop.

Young surgeons must acquire different left-hand movements to shift from the divide-and-conquer technique to phaco-chop. Herein, we report our new technique, which would enable effortless mastery of the phaco-chop technique. The basis of our technique is the generation of a new lens equator inside the capsulorhexis by drilling two holes. Drilling two holes adjacent to the capsulorhexis mimics a new lens equator; the lens appears smaller, enabling surgeons-in-training to master the phaco-chop more safely and easily without pushing the lens posteriorly.

## Methods

Three surgeons in their first year of training (fellows A, B, and C) performed the phaco-chop technique. Before learning this technique, two attending surgeons had previously experienced grooving the nucleus, whereas the third surgeon had not. That is, the third surgeon directly acquired knowledge of the phaco-chop technique after repeatedly practicing the two-hole method.

We compared the cumulative dissipated energy (CDE) and case ultrasound time (UST) with three phacoemulsification techniques (divide-and-conquer, two-hole method, and phaco-chop) among the three young surgeons. We collected data of their first 8 cases and analyzed a total of 64 cases: 16, divide-and-conquer; 24, two-hole method; 24, phaco-chop.

### Surgical technique

In this study, the Constellation Vision System (Alcon Laboratories, Inc., Fort Worth, TX, USA) was used. Phacoemulsification was performed with a 45$^\circ$ Kelman Mini tip under the flow mode. For the two-hole technique, the phaco-tip was buried into the nucleus about 3/4 depth of the lens adjacent to the capsulorhexis; this step was repeated after rotating the nucleus by 180$^\circ$ to make two holes on the nucleus. We used sleeve irrigation port as a reference for the depth. To avoid damaging the anterior capsule, the phaco-tip was carefully placed inside the capsulorhexis and then pulled until the phaco-tip stood as vertically as possible. Phacoemulsification power was maintained low at 20% to reduce the risk of damage to the posterior capsule in the early stages of the surgery. Power can be increased as the surgeons-in-training get used to this technique. Other settings for drilling holes were vacuum 220–250 mmHg, aspiration flow rate 30 mL/min, phaco power 20–40%, and infusion bottle height 85 cmH_2_O.

Before drilling the two holes on the nucleus adjacent to the capsulorhexis, the lens nucleus was separated from the capsule to enable its smooth rotation in the capsular bag. Hydrodissection and removal of the cortex from the periphery were performed sufficiently beforehand.

**Fig. 1:**
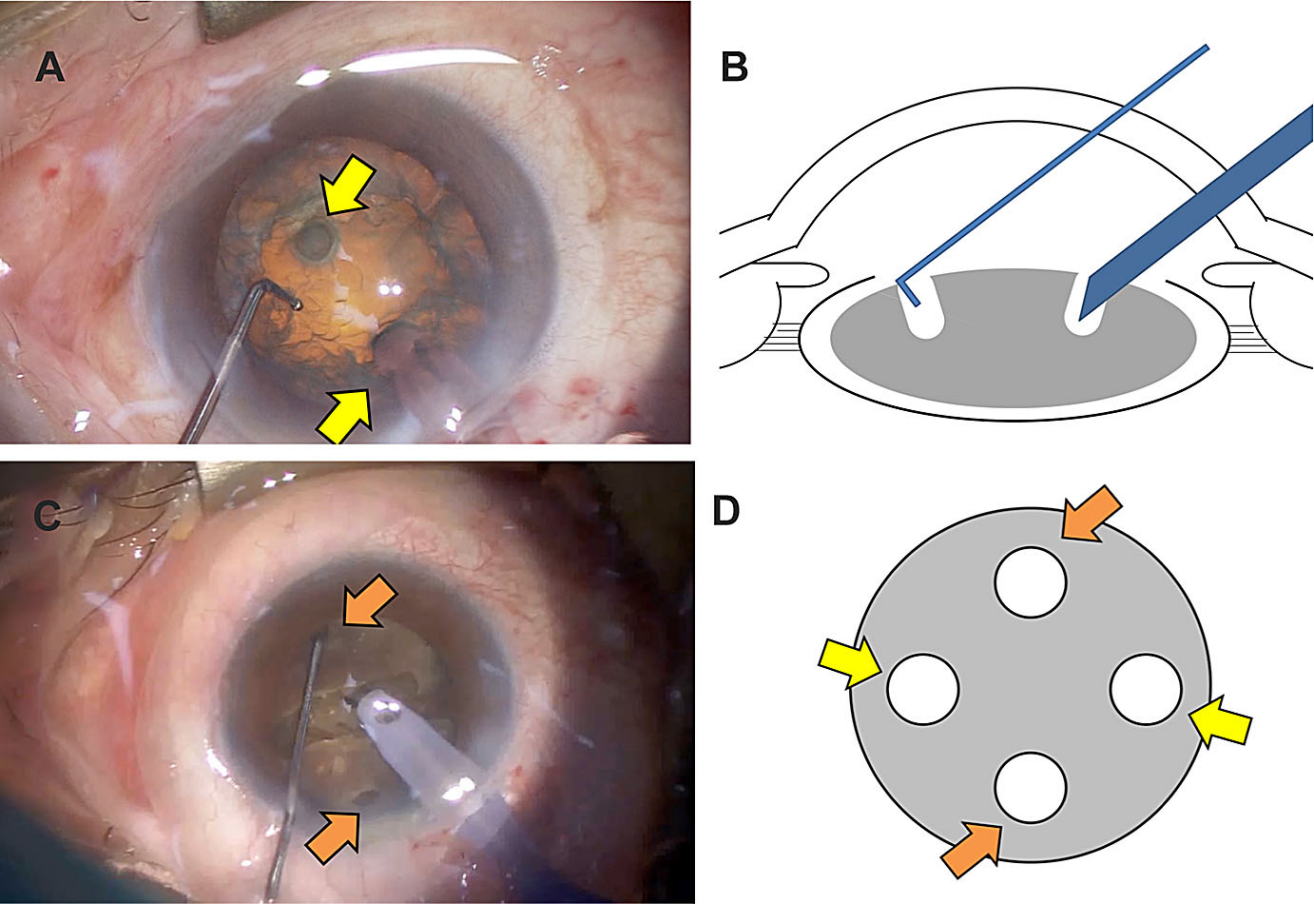
**a** Top view of the chopper and the phaco-tip being buried into the two holes (yellow arrows) adjacent to the capsulorhexis. **b** Side view of the placement of the chopper and phaco-tip as described in **a**. **c** and **d** Two holes (orange arrows), additional to the original holes (yellow arrows), are drilled adjacent to the capsulorhexis. Drilling four holes enables effective nuclear fracturing in cases with a hardened nucleus

The phaco-tip was inserted into one of the holes. Thereafter, the chopper was inserted deeply into the other hole (Fig. 1a, b). The two instruments were moved back and forth relative to each other until the nucleus cracked into two separate pieces. The supplementary videos (Video 1, 2) show a shortened version of the procedure used in this study.

After drilling the two holes on the nucleus adjacent to the capsulorhexis, nucleofractis and quadrant removal were performed under the following settings: phaco power 0 %, torsional power 70 %, vacuum 380 mmHg, aspiration flow rate 40 mL/min, and infusion bottle height 90 cmH_2_O.

## Results

The young practicing surgeons could eventually perform the standard phaco-chop, with the two-hole method, more easily and successfully.

The drilling of holes enabled deep and effortless impaling of the nucleus.

Almost all these maneuvers were performed inside the capsulorhexis; therefore, the risk of laceration was minimized. Despite the small pupil size or presence of IFIS, the first-year fellows successfully completed the procedure. In a few cases, we found round continuous notches of the anterior capsulorhexis in the two-hole group because of drilling over the anterior capsule. We did not count this as a complication because the continuity and the integrity of capsulorhexis were not altered. Among the 64 cases, there were four intraoperative complications: one small zonular dehiscence each in the divide-and-conquer group and in the two-hole group, one posterior capsule rupture with vitreous loss in the two-hole group, and one radial capsulorhexis tear in the phaco-chop group. The case with posterior capsule rupture was excluded during the calculation of the average, and the next consecutive case was considered in place of the excluded case. There were no early postoperative complications.

**Fig. 2:**
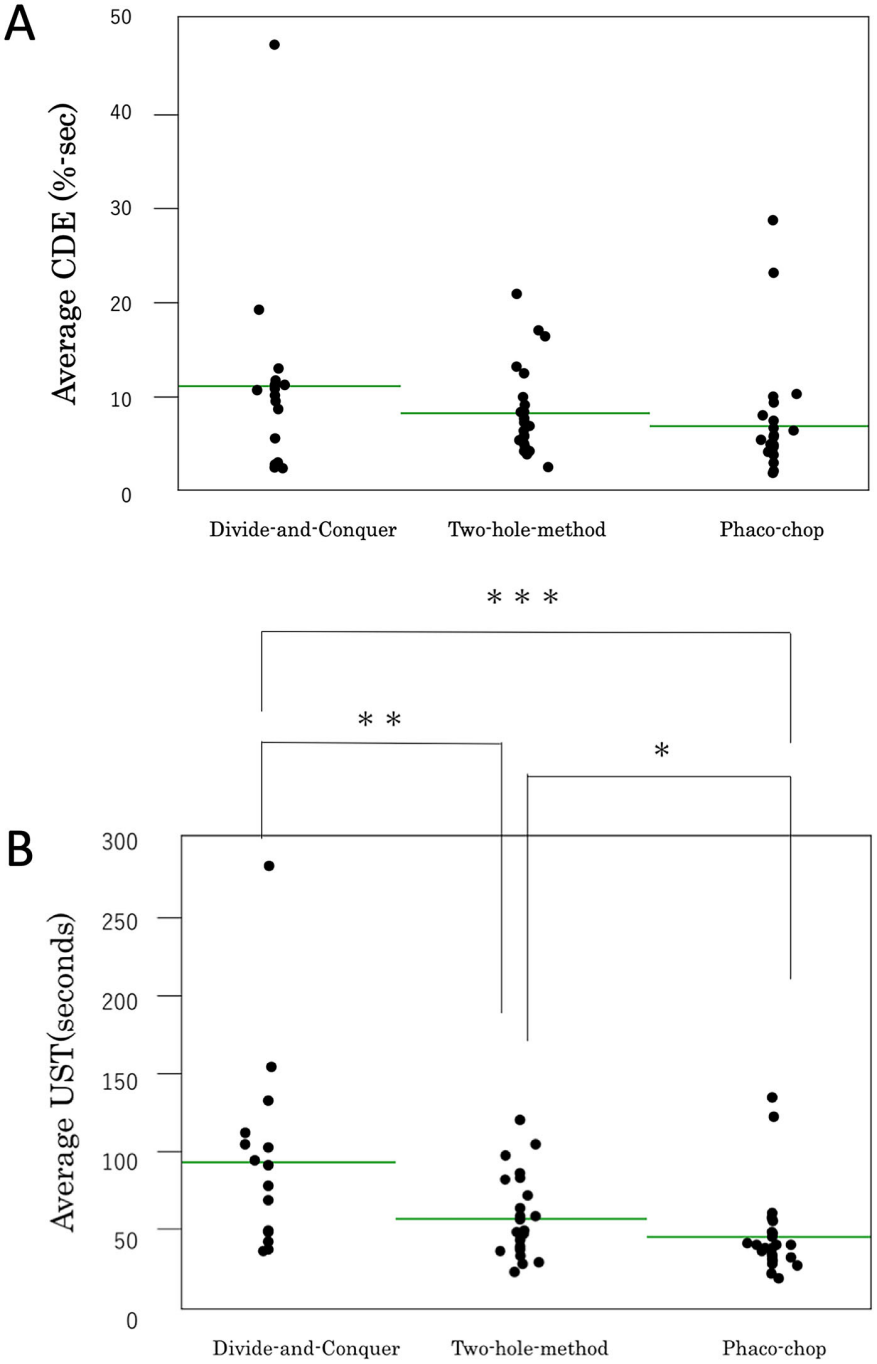
**a** Comparison of cumulative dissipated energy among the three techniques. A significant difference was not observed among the groups (*P* = 0.0538) with the non-parametric Kruskal–Wallis test. **b** Comparison of the loss of ultrasound time among the three techniques. There was a significant difference among the groups (*P* = 0.0008) with the non-parametric Kruskal–Wallis test. **Divide-and-conquer vs. two-hole (*P* = 0.0219). ***divide-and-conquer vs. phaco-chop (*P* = 0.0004). *two-hole vs. phaco-chop (*P* = 0.0477)

Figure 2a shows the analysis of CDE data for each technique. CDE was 11.4%-sec for divide-and-conquer, 8.5%-sec for the two-hole technique, and 7.2%-sec for phaco-chop. Although the comparison between each group showed a significant difference between the divide-and-conquer and phaco-chop techniques (*P* = 0.0371), a three-group comparison with the non-parametric Kruskal–Wallis test did not reveal any significant differences (*P* = 0.0538).

On the contrary, significant differences were seen in UST (*P* = 0.0008; Fig. 2b) among the three techniques. UST was 91.4 s for divide-and-conquer, 55.8 s for the two-hole technique, and 44.5 s for phaco-chop. Significant differences were also observed in two-group comparisons: Divide-and-conquer vs. two-hole (*P* = 0.0219); divide-and-conquer vs. phaco-chop (*P* = 0.0004); and two-hole vs. phaco-chop (*P* = 0.0477).

## Discussion

To minimize tissue damage and complications, the phaco power must be reduced and used efficiently. The phaco-chop technique is one of the most effective methods for nuclear fracturing and requires less time for ultrasound sculpting. In this study, we aimed for the surgeons-in-training to master the phaco-chop technique steadily and quickly. While focusing on enabling mastery of the first chopping, we envisioned this technique of drilling two holes adjacent to the capsulorhexis that would mimic a new lens equator for easier first chopping. Using this approach, young surgeons who are attempting the technique for the first time can efficiently remove the initial segment.

There are only a few studies about the different phacoemulsification techniques efficiency among surgeons-in-training. Wong et al. reported an average UST with phaco-chop of 72 s, which was less than the average UST with divide-and-conquer (124 s) [4]. Although the equipment used and the conditions were different, our study also shows an advantage of a shorter UST with the phaco-chop technique.

Coppola et al. compared the efficiency of surgeons-in-training when performing the divide-and-conquer and stop-and-chop techniques. They reported that even for surgeons-in-training, the stop-and-chop technique was more efficient for advanced cataracts and encouraged a switch from divide-and-conquer to stop-and-chop [9].

Gross et al. compared the efficiency of nuclear disassembly among resident surgeons while using the divide-and-conquer technique and the pop-and-chop technique, [10] which was described by Pandit and Oetting in 2003 [11]. The pop-and-chop technique enables an easy first crack after partial extracapsular prolapse of the nucleus prior to the initial chop. They reported a CDE of 15.9%-sec with divide-and-conquer and 8.6%-sec with pop-and-chop. The average surgical time was 31 min and 17.8 min, respectively. Although pop-and-chop is a more time- and energy-efficient technique than divide-and-conquer for nucleofractis in the case of novice resident surgeons, the first chop is performed on the bag space. In contrast, our two-hole technique is performed in the bag space, which is the same as the standard and other advanced techniques. Regarding efficiency, although the equipment and the conditions may be different, our two-hole technique shows similar CDE to that of the pop-and-chop technique, and our surgeons-in-training could migrate the techniques to the standard in-the-bag phaco-chop technique effortlessly.

This method can also be implemented by experienced surgeons. Drilling four holes adjacent to the capsulorhexis enables effective nuclear fracturing in cases with a hardened nucleus (Fig. 1c, d). First, through two of the four holes drilled in opposite angles, the nucleus is split into two pieces according to the conventional phaco-chop technique. Thereafter, using the other two holes, each hemi-nucleus is chopped into two smaller pieces, resulting in the nucleus splitting into four pieces. This variation enables more effective nucleus fracturing than the conventional methods of grooving denser cataracts.

In cases of a temporal approach for the left eye, surgeons may rotate the surgical microscope and position themselves at the temporal side of the patient. However, drilling holes enables a regular superior approach more easily without requiring a change in their sitting positions.

Thus, these holes adjacent to the capsulorhexis, which mimic a smaller lens equator, enable more efficient phaco-chopping not only for surgeons-in-training but also for experienced surgeons.

Although we observed a difference in the UST, the sample size and power of this study were insufficient for conclusive results. Difficulty in the preparation of cataracts of the same type and density among these three surgeons might have reduced the accuracy of the study by introducing sample bias. Surgeons should have improved their skills after every case. For accurate analysis, all three surgeons should have started learning these three techniques simultaneously. Therefore, further studies with a larger sample size are needed to validate our findings that our technique reduces both energy levels and UST when compared with conventional techniques like the divide-and-conquer method.

## Conclusion

This study aimed to enable mastery of the phaco-chop technique by surgeons-in-training steadily and quickly. Although no significant difference was found among the techniques in the CDE, there was a significant difference in the UST (*P* < 0.0 5).

Our results suggest that the two-hole method may improve efficiency among surgery-residents-in-training. Owing to flaws in the design of the study, possible bias, and lack of statistical power, further studies with a larger sample size may be needed to examine the use of the two-hole method in nucleofractis techniques.

## Supplementary Information

Below is the link to the electronic supplementary material.

Supplementary file1 (MP4 35736 KB)

Supplementary file2 (MP4 25137 KB)
